# Supplementary material for: A multi-dimensional fusion strategy similarity measure method for patent application technology disclosure document
Source: PLoS One. 2023 Oct 18;18(10):e0293091. doi: 10.1371/journal.pone.0293091 (PMC10584106; doi:10.1371/journal.pone.0293091)
Supplement: Attachment — Submitted filename: Response to Reviewers.docx [file pone.0293091.s008.docx]

Dear academic editor and reviewers:

Thanks for the careful review of our manuscript. Your recognition and suggestions are crucial for us. We have revised the manuscript carefully, and please find the following detailed reply and actions to your comments and suggestions one by one:

Academic editor:

1. Please ensure that your manuscript meets PLOS ONE's style requirements, including those for file naming. The PLOS ONE style templates can be found at <https://journals.plos.org/plosone> /s/file?id=wjVg/PLOSOne_formatting_sample_main_body.pdf and [https://journals.plos.org/ploso ne/s/file?id=ba62/PLOSOne_formatting_sample_title_authors_a](https://journals.plos.org/plosone/s/file?id=ba62/PLOSOne_formatting_sample_title_authors_a)ffiliations.pdf.

Reply:Thanks for this reminder, we do have some problems with the format of the manuscript we submitted before.

Action:We have carefully read the submission specification of PLOS One journal and the pdf file you provided, and systematically revised and improved our manuscript according to the requirements therein.

2. In your Data Availability statement, you have not specified where the minimal data set underlying the results described in your manuscript can be found. PLOS defines a study's minimal data set as the underlying data used to reach the conclusions drawn in the manuscript and any additional data required to replicate the reported study findings in their entirety. All PLOS journals require that the minimal data set be made fully available.

Reply:Thanks for this comment. We have uploaded part of the supporting dataset of the experimental results of this paper in the supplementary materials when we submitted our manuscript before. The remaining datasets will be supplemented during the upload process for our revised manuscript.

Action:We have supplemented the smallest datasets previously uploaded, and we have added a section of the data availability statement in our paper. The latest datasets will be able to be used to draw all the conclusions in our paper.

Reviewer #1: This paper proposes a multi-dimensional fusion strategy similarity measure method for patent application technology disclosure documents by using many machine-learning-based similarity measurement approaches. The paper targets an important topic, but there are several problems with this paper.

Reply:Thank you for your professional review of our manuscript. As you are concerned, there are several problems that need to be addressed. According to your precious suggestions, we have revised the manuscript carefully, the detailed reply and actions are listed below:

1. The introduction does not discuss the specific challenges faced by the task of measuring the disclosure documents, which is very important for your paper. First, we cannot know why the existing deep learning-based text similarity analysis cannot solve the current task. Second, we cannot know what motivated you to design your model, and why your model is effective.

Reply:Thanks for this comment, it should be discussed specifically in the introduction part.

Action:We have added an analysis of the characteristics of the patent application technology disclosure documents in the introduction section of the article. Based on the analysis of features, the problems and challenges of existing methods in solving the similarity measurement of the above documents are discussed, and supplementary experiments are added in subsequent chapters to prove the effectiveness of the proposed algorithm.

2. The model considers character similarity, which has been shown by many papers to be weaker than word vector analysis, so what is the point of its existence, and has it been followed up with ablation experiments to prove it?

Reply:Thanks for your question in this section. It is true that in most cases, the analysis of character similarity is indeed weaker than that of word vectors, but due to the unique characteristics of patent application technical disclosure documents, character similarity can play a positive role in the overall measurement of similarity of the above documents.

Action:We have added an analysis and discussion of the above issues in the module on character similarity.

3. All of the analysis approaches are based on machine learning or simple statistical analysis; I cannot find any innovations in modeling.

Reply:Thank you for pointing this out. Although we have not made structural innovations for related models, we have put forward new insights in the perspective and method of text similarity analysis, such as optimizing the computational bias of similarity by introducing reconstruction and scoring sections, mapping text vectors to dot matrix and image spaces to analyze and calculate their similarity, etc. And we are planning to try to combine the proposed method with the feature extraction module of the deep learning model to achieve better performance.

4. Most of the datasets used in the experiment are designed for common text analysis. Tests against these datasets are not meaningful for the scenarios in this paper, and the proposed method is significantly weaker than deep-learning-based methods. And, the only effective dataset is not open-source. I think the patent application technology disclosure documents have already been released after they are submitted, so why not open source these documents?

Reply:Thanks for this comment. Our experiments on the common datasets are intended to demonstrate the feasibility of the proposed method in the similarity evaluation tasks, although there is a certain gap in performance compared to deep learning models, it also has certain advantages. As for the problem of patent application technology disclosure document dataset, we have obtained an open source license for it.

Action:We have collated all remaining datasets related to the output of the results of the experiments in the paper and submitted them as supplementary material along with the revised manuscript.

5. Without doing comparison experiments on the only valid dataset, we cannot know the superiority of the proposed method over existing text similarity analysis methods.

Reply:Thank you for pointing this out. This is indeed an indispensable part of the experiments to prove the superiority of the proposed method in our paper.

Action:We have supplemented this part of the experiment and discussed it based on the results of the comparative experiment.

Reviewer #2: The proposed similarity assessment method of patent application technical disclosure documents under a multi-dimensional fusion strategy is novel. The proposed method significantly improves the discriminant accuracy by about 10% compared with the traditional vector semantic model. The proposed method is evaluated using four published text similarity matching datasets (containing 0-5 or 0/1 labels) and a set of patent application technology disclosure documents.

Reply:Thanks for the careful review of our manuscript. We sincerely appreciate your recognition and encouragement. We have revised the manuscript carefully, the detailed reply and actions are listed below:

1. It would be nice to add an overview figure to the paper to represent the proposed multidimensional fusion strategy.

Reply:Thanks for this suggestion, we accept it thoroughly.

Action: We have added an overview figure of the multi-dimensional fusion strategy similarity measure method proposed in this paper in the methodology section of the manuscript.

2. The proposed method can be compared to the discrimination ability of a lightweight deep learning model without training. However, the deep learning model outperforms the proposed method by a large margin. Therefore, this paper is limited in terms of usability. Furthermore, it is expected that higher performance can be achieved by applying multi-dimensional fusion strategies to deep learning models.

Reply:Thank you for pointing this out. The evaluation performance of our proposed method on the common datasets of text similarity does have a certain gap with that of the deep learning models, but we have added a supplementary experiment to confirm that the proposed method has strong advantages for the similarity evaluation task of patent application technology disclosure document data. And we are planning to try to combine the proposed method with the feature extraction module of the deep learning model according to your suggestion in the follow-up study to achieve better performance.

3. It may be difficult for readers without a background in the field to understand, so it is necessary to explain in detail the characteristics of patent documents and multidimensional fusion strategies.

Reply:Thanks for this suggestion, it is indeed necessary.

Action:We have added a detailed description of the characteristics of patent application technology disclosure document and a brief supplement to the multi-dimensional strategy in the introduction of the manuscript.

4. The paper does not provide a detailed discussion of the limitations of the proposed method and areas for future research.

Reply:Thank you for pointing this out. We did ignore this part.

Action:We have added a discussion of the limitations of the proposed method and an outlook for future research directions at the end of the conclusions section.
