# Supplementary material for: A multi-dimensional fusion strategy similarity measure method for patent application technology disclosure document
Source: PLoS One. 2023 Oct 18;18(10):e0293091. doi: 10.1371/journal.pone.0293091 (PMC10584106; doi:10.1371/journal.pone.0293091)
Supplement: Attachment — Submitted filename: Response to Reviewers.docx [file pone.0293091.s009.docx]

Dear academic editor and reviewers:

Thanks for the careful review of our revised manuscript. Your recognition of our revised content and the proposed new suggestions are important for the further refinement of our manuscript. We have revised the manuscript carefully, and please find the following detailed reply and actions to your comments and suggestions one by one:

Reviewer #1: Thanks so much for the revision, and many of the problems are addressed. But, several problems are raised by the revision, which should also be addressed.

**Reply:**Thanks for your recognition of our modification work. According to your precious suggestions for the revised manuscript, we have revised the manuscript again carefully, the detailed reply and actions are listed below:

1. It is reasonable that refining an abstract can exclude the interference of too long text and irrelevant text. How to refine the abstract should be the focus of this paper. The extraction method is based on the rules of extraction; the innovation and effectiveness of its method should be evaluated and compared with the abstract extraction method based on deep learning.

**Reply:**Thank you for pointing this out. The abstract is indeed the most important part of an article, which can make people intuitively understand the research topics and innovation points of this article. In our study, the refinement of the key content of the patent text is achieved through word segmentation, screening, reconstruction and scoring in text preprocessing. For our method, the input is a preprocessed character or word vector, while for the deep learning model, the input is the original patent text. Although the ultimate goal is to assess the similarity of patents, the realization process does have certain differences. As you mentioned, the extraction method based on deep learning is relatively abstract, making it difficult to visually observe the process and results of deep learning models extracting patent text features. Therefore, it is not possible to make a simple comparison between the content extraction results of the deep learning models and ours. Moreover, due to the significant differences in the similarity calculation methods between the deep learning models and ours, it is difficult to determine the impact of these two extraction results on the final similarity calculation. However, this part does give us a new idea for follow-up research, which can visualize the feature extraction process and the final category determination mechanism of the deep learning models to achieve the comparison of the content refining effect and the impact on the subsequent evaluation results.

**Action:**We have added a quantitative description of the experimental results of our method and other methods on different datasets in the abstract section of the article. And in the methodology and discussion section of the article, a more detailed discussion and effectiveness verification were conducted on the feature extraction method proposed in this article.

2. It makes sense to use character matching because there are special characters and terms, but it should be supported by ablation experiments in the evaluation.

**Reply:**Thanks for this comment, we agree with your [perspective](javascript:;).

**Action:**We have added corresponding ablation experiment to better support our viewpoint.

3. The authors say the contribution of modeling is optimizing the computational bias of similarity by introducing reconstruction and scoring sections, but there are no ablation experiments to support it.

**Reply:**Thanks for this comment. The ablation experiment should be added to demonstrate the effectiveness of these two sections.

**Action:**We have conducted a comprehensive ablation experiment on the two proposed improvements (reconstruction and scoring) and analyzed the experimental results to support our viewpoint.

4. For the comparison based on the related document dataset, only giving accuracy is not enough. The precision and F1 score should also be given.

**Reply:**Thanks for this suggestion, we accept it thoroughly.

**Action:**We have re-evaluated the test results of each model and added three new indicators: precision, recall, and F1 score for a more comprehensive evaluation.

Reviewer #2: The authors have responded to reviewer comments and made revisions to address the concerns raised.

**Reply:**Thanks for the careful review of our revised manuscript. We sincerely appreciate your recognition of our modification work. We have carefully considered your concerns and further improved the manuscript.

1. Readers without a background in the field may find it difficult to understand the nature of patent documents and multidimensional fusion strategies. For the benefit of the reader, it would be helpful to smooth out several sentences and make the proposed methods and experiments reproducible.

**Reply:**Thanks for this suggestion, this is indeed a problem that deserves our consideration and resolution.

**Action:** We have carefully reviewed and refined the language throughout our paper, provided a more accurate description of the proposed method and supplemented with corresponding ablation experiments to further verify the effectiveness of our method.
